# Initial experience with zero-fluoroscopy pulmonary vein isolation in patients with atrial fibrillation: single-center observational trial

**DOI:** 10.1038/s41598-024-67183-7

**Published:** 2024-07-15

**Authors:** Dalma Torma, Kristof Janosi, Dorottya Debreceni, Botond Bocz, Mark Keseru, Tamas Simor, Peter Kupo

**Affiliations:** Medical School, Heart Institute, University of Pecs, Ifjusag utja 13., 7624 Pecs, Hungary

**Keywords:** Atrial fibrillation, Ablation, Pulmonary vein isolation, Zero-fluoroscopy, Cardiology, Interventional cardiology

## Abstract

Pulmonary vein isolation (PVI) stands as a widely practiced cardiac ablation procedure on a global scale, conventionally guided by fluoroscopy. The concurrent application of electroanatomical mapping systems (EAMS) and intracardiac echocardiography offers a means to curtail radiation exposure. This study aimed to compare procedural outcomes between conventional and our initial zero-fluoroscopy cases in patients with paroxysmal or persistent atrial fibrillation (AF), undergoing point-by-point PVI. Our prospective observational study included 100 consecutive patients with AF who underwent point-by-point radiofrequency PVI. The standard technique was used in the first 50 cases (Standard group), while the fluoroless technique was used in the subsequent 50 patients (Zero group). The zero-fluoroscopy approach exhibited significantly shorter procedural time (59.6 ± 10.7 min vs. 74.6 ± 13.2 min, p < 0.0001), attributed to a reduced access time (17 [16; 20] min vs. 31 [23; 34.5] min, p < 0.001). Comparable results were found for the number of RF applications, total ablation energy, and left atrial dwelling time. In the Zero group, all procedures were achieved without fluoroscopy, resulting in significantly lower fluoroscopy time (0 [0; 0] sec vs. 132 [100; 160] sec, p < 0.0001) and dose (0 [0; 0] mGy vs. 4.8 [4.1; 8.2] mGy, p < 0.0001). The acute success rate was 100%, with no major complications. Zero-fluoroscopy PVI is feasible, safe, and associated with shorter procedure times compared to the standard approach, even in cases without prior experience in zero-fluoroscopy PVI.

## Introduction

Atrial fibrillation (AF) is the most common sustained cardiac arrhythmia in adults. The indication for rhythm control strategy is to reduce AF-related symptoms and improve quality of life according to the most recent guidelines published by European Society of Cardiology^1^. Catheter ablation is the recommended treatment of symptomatic, drug-refractory AF which is superior to antiarrhythmic drugs (AAD) for the maintenance of sinus rhythm^2–4^. The main aspect of AF ablation procedures for patients experiencing symptomatic paroxysmal or persistent AF resistant to AAD therapy is the electrical isolation of the pulmonary veins (PVs). This is by far the most commonly performed cardiac ablation procedure worldwide^1,5,6^.

Traditionally, these procedures are guided by fluoroscopy, exposing both patients and medical staffs to considerable and potentially hazardous ionizing radiation. During AF ablation procedures, the typical exposure to fluoroscopy is 15 mSv surpassing levels observed in other ablation procedures and associated with an elevated risk of fatal and non-fatal cancer, estimated at 1 in 750 for men at the age of 50 years^7^.

The widespread adoption of electroanatomical mapping systems (EAMS) has facilitated a significant reduction in fluoroscopy exposure during electrophysiology (EP) procedures. Currently, the prevalence of fluoroscopy-free catheter ablation is increasing, particularly in the context of supraventricular tachycardia ablations^8–10^. Nevertheless, a recently published meta-analysis provides evidence supporting the effective and secure implementation of the zero fluoroscopic strategy in AF ablation procedures^6^.

The accumulating evidence has prompted our institution to initiate the adoption of zero-fluoroscopy point-by-point pulmonary vein isolation (PVI) procedures. In our prospective observational trial, our objective was to compare procedural data derived from the initial 50 cases of zero-fluoroscopy PVIs with the data from 50 PVIs performed conventionally before.

## Methods

### Study population

In our prospective observational, single-center study, we enrolled 100 consecutive patients undergoing point-by-point PVI procedures for paroxysmal or persistent AF. The first 50 patients underwent standard PVI procedures guided by EAMS, in addition to intracardiac echocardiography (ICE) and fluoroscopy (Standard group). Subsequently, for cases 51–100, PVI was conducted without the use of fluoroscopy, relying solely on EAMS and ICE guidance (Zero group). Exclusion criteria included (a) prior PVI procedures, (b) additional ablations beyond PVI (including any left or right atrial ablations), and (c) age under 18 years. All transseptal punctures (TSP) and PVIs were performed by the same experienced electrophysiologist with great experience in right-sided fluoroless SVT ablations, but no relevant prior experience in zero-fluoroscopy TSP or zero-fluoroscopy PVI. The femoral vein punctures and catheter placements preceding TSP were conducted by two EP fellows. The trial protocol adheres to the Declaration of Helsinki, and the study received approval from the regional ethics committee (Approval number: 9409). Written informed consent was obtained from all patients participating in the study.

### Catheter placement in the Standard group and fluoroscopy plus ICE-guided transseptal puncture

During the procedures, conscious sedation was induced with fentanyl ± midazolam. After administering local anesthesia and conducting a double left sided and a single right sided femoral venous puncture guided by vascular ultrasound, a 10F ICE catheter was advanced and the placement of a decapolar steerable catheter (Dynamic Deca, Bard Electrophysiology, Lowell, MA, USA) was positioned under fluoroscopy guidance in the coronary sinus (CS) served as an anatomical landmark for TSP.

The transseptal sheath (SL0, Abbott Laboratories, Chicago, IL, USA) and dilator are progressed over the guidewire towards the superior vena cava (SVC) using fluoroscopy (anteroposterior projection). After removing the guidewire, a Brockenbrough needle (BRK-1 XS, Abbott Laboratories, Chicago, IL, USA) was inserted. Then, the stylet of the needle was removed, and a screw syringe was connected to the needle. The pulling back maneuver was started at 4 o’clock position. The needle and the sheath were pulled back caudally under fluoroscopy guidance until they were visualized in the inferior portion of the SVC and after that at the fossa ovalis. Tenting was carefully monitored by ICE. Following the TSP, the SL0 was replaced with a visualizable steerable sheath (VIZIGO, Biosense Webster Inc., Irvine, CA).

### Catheter placement in the Zero group and fluoroscopy-free transseptal puncture

**Figure 1:**
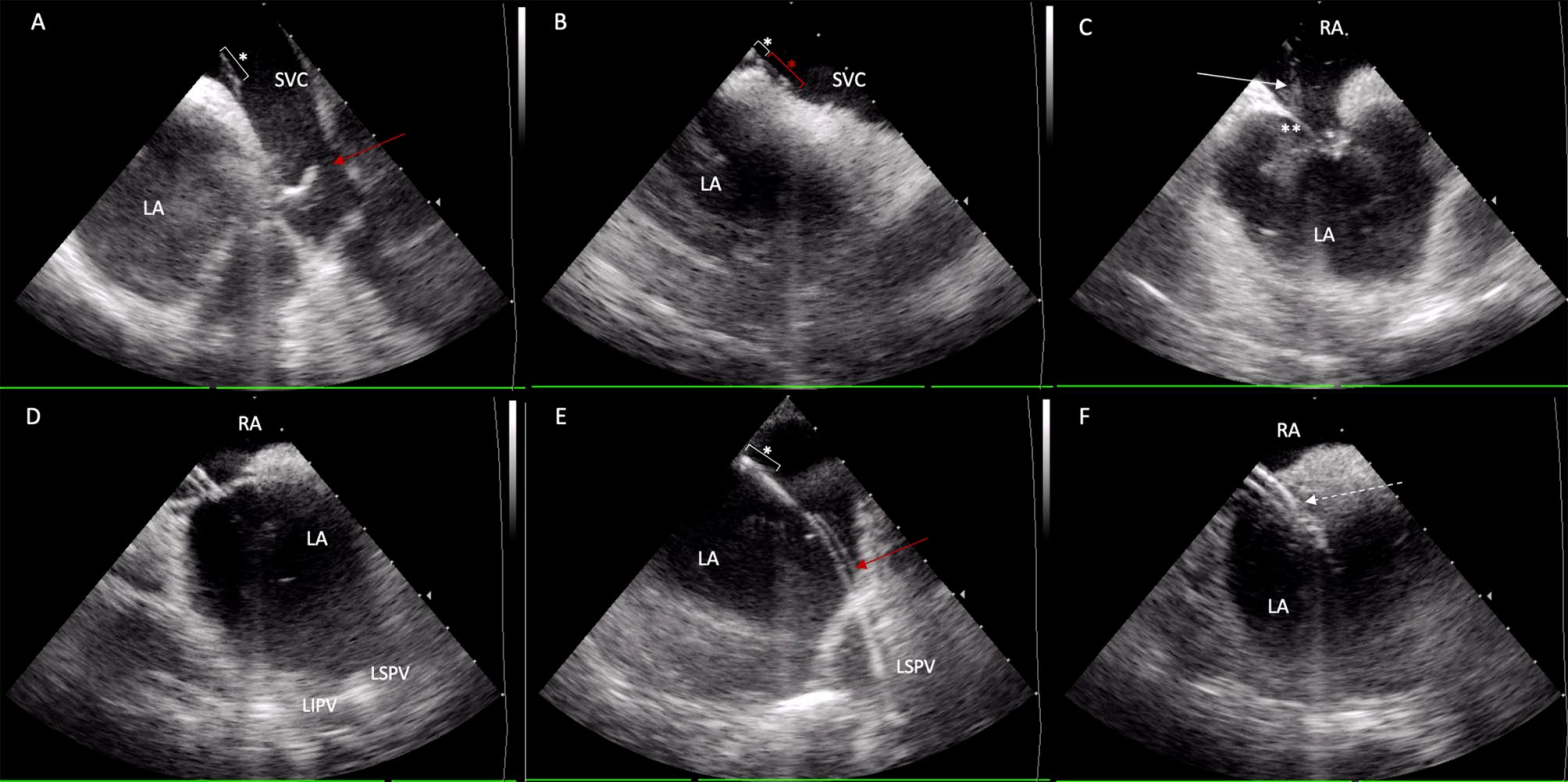
Llustrative intracardiac echocardiography (ICE) images capturing crucial moments in the zero-fluoroscopic transseptal puncture procedure. Panel (**A**) shows the guidewire (red arrow) and the fixed-cure long sheath (white asterisk) located in the superior vein cava. In Panel (**B**) the guidewire is switched to a transseptal needle (red asterisk) visible at the end of the fixed sheath (white asterisk), both still located in the SVC. Panel (**C**)—by pulling the sheath and the needle down (white arrow), they appear in the fossa ovalis (double white asterisk) creating the ”tenting” phase of the transseptal puncture. After transseptal puncture, in Panel (**D**) the sheath is visualized, inserted into the LA. Panel (**E**) shows the guidewire introduced in the LSPV, thus in the next step the fixed sheath can be replaced to a steerable sheath (dashed arrow), as in Panel (**F**). ICE, intracardiac echocardiography; SVC, superior vena cava; LA, left atrium; RA, right atrium; LSPV, left superior pulmonary vein; LIPV, left inferior pulmonary vein.

While in the Standard group the decapolar catheter was mainly used as an anatomical landmark for TSP, to simplify procedures in the Zero group, the use of a decapolar catheter was omitted. After the double femoral venous puncture, insertion of a 10F ICE probe and a guidewire ensued. Following a double femoral venous puncture, a 10F ICE probe and a guidewire were inserted. Subsequently, a single long J-wire was advanced into the superior vena cava, and its positioning was verified by ICE deflection posteriorly and slight right rotation. An SL0 sheath was then guided over the wire into the SVC, with subsequent removal of the guidewire. After meticulous confirmation of the distal end of the dilator using ICE, a BRK-1 needle was introduced into the sheath, and the entire system was retracted under ICE guidance. The distal end of the SL0 sheath was precisely positioned at the fossa ovalis, and the transseptal puncture was executed using the transseptal needle. Once the dilator of the SL0 reached the left atrium, the needle was exchanged for the guidewire, which was navigated to either of the left PVs. The position of this guidewire was verified by ICE located in the right ventricle. Subsequently, the SL0 sheath was advanced into the left atrium and exchanged for a VIZIGO steerable sheath. Figure 1 illustrates the procedural steps of ICE-guided zero-fluoroscopy TSP.

### Left atrial workflow

A fast anatomical map of the left atrium was created by the Pentaray catheter (Biosense Webster Inc., Diamond Bar, CA, USA), utilizing support from the CARTO electroanatomical mapping system (Biosense Webster Inc., Diamond Bar, CA, USA). Following the creation of the anatomical map, the multipolar mapping catheter was replaced by a radiofrequency ablation catheter (Navistar Thermocool SmartTouch ST NAV, Biosense Webster Inc., Diamond Bar, CA, USA), which was set in a power-controlled mode with a maximum power of 45 W for the anterior wall and 40 W for the posterior wall, using a maximum temperature of 43 °C. During radiofrequency (RF) ablations, the CARTO VISITAG™ Module was utilized, featuring a minimum stability time of 4 s and a maximum location stability range of 2.5 mm. The Visitag Surpoint (ablation index) guided the procedure, with targets set at 350 for the posterior wall and 450 for the anterior wall. To maintain a target interlesion distance below 5 mm, a point-by-point ablation technique was implemented, with continuous real-time monitoring of contact force (CF) and impedance. CF was carefully maintained within the range of 5–15 g throughout the ablation process. Following the completion of ablation circuits encircling the PVs, the ablation catheter was transitioned to Pentaray for the verification of electrical isolation of the PVs. In instances where initial isolation was not achieved, additional ablations were undertaken before revalidation.

Intravenous unfractionated heparin was promptly administered following femoral vein puncture, and an activated clotting time exceeding 300 s was maintained throughout the entire procedure. The procedural objective for the ablation was considered achieved when isolation was confirmed in all PVs. In adherence to our institutional protocol, only PVI was undertaken, even in cases of persistent AF.

### Study endpoints

The primary endpoint of this study was the skin-to-skin procedure time, which was defined as the duration from the femoral vein puncture to the removal of the last catheter. The procedure time was divided into distinct segments, including the time from puncture to the initiation of left atrial mapping (defined as access time), mapping time, the interval between the first and last RF application, and validation time. Additionally, we documented the left atrial dwelling time, the number of RF applications, total ablation time, and total ablation energy. The fluoroscopy duration and dose were automatically computed using data from the fluoroscopy system. Furthermore, we recorded the rate of first pass isolation and potential major complications during the periprocedural period, such as vascular complications, pericardial effusion/tamponade, and atriooesophageal fistula. Hematoma was classified as a major vascular complication if it fulfilled the Bleeding Academic Research Consortium (BARC) criteria of type 2 or higher. This includes cases necessitating nonsurgical medical intervention by a healthcare professional, resulting in prolonged hospitalization or an increased level of care, or prompting further evaluation^11^.

### Statistical analysis

Data were analyzed for normal distribution using the Kolmogorov–Smirnov goodness-of-fit test. Continuous data were expressed as mean ± standard deviation (SD) or median (interquartile range, IQR), as appropriate, while categorical variables were represented as absolute numbers and percentages. Comparative analyses employed the chi-square test, t-test, and Mann–Whitney U test, as applicable. Statistical significance was defined as a p-value < 0.05 for all analyses. The statistical procedures were carried out using SPSS 28 software (SPSS, Inc., Chicago, IL, USA).

### Ethical approval

The trial protocol adheres to the Declaration of Helsinki, and the study received approval from the regional ethics committee. All persons gave their informed consent prior to their inclusion in the study.

## Results

A total of 100 patients were included in the study. As detailed in the “Methods” section, the first 50 patients underwent standard PVI procedures, while for cases 51–100, PVI was conducted without the use of fluoroscopy. No exclusion criteria were applied to patients during or after the procedure. There was no observed significant difference in baseline characteristics between the groups in the study population (male sex: 50% vs. 46%, p = 0.61; age: 69.5 (58; 72.3) years vs. 68 (57.5; 73) years, p = 0.59, as shown in Table 1).

**Table 1:** Study and patients’ characteristics of the included trials.

	Standard group (n = 50)	Zero group (n = 50)	p value
Age, years	69.5 (58; 72.3)	68 (57.5; 73)	0.59
Male (%)	25 (50)	23 (46)	0.61
CHA_2_DS_2_-VASc score	2.36 ± 1.47	2.27 ± 1.43	0.37
HASBLED score	1.98 ± 1.19	1.78 ± 1.01	0.19
Body mass index	28.7 ± 3.10	28.9 ± 3.2	0.71
Hypertension (%)	42 (84)	40 (80)	0.75
Diabetes mellitus (%)	9 (18)	15 (30)	0.14
Heart failure (%)	6 (12)	5 (10)	0.77
Coronary artery disease (%)	16 (32)	10 (20)	0.19
Chronic kidney disease (%)	3 (6)	3 (6)	1
Prior stroke/TIA (%)	6 (12)	2 (4)	0.14
Paroxysmal atrial fibrillation (%)	30 (60)	30 (60)	1
LA diameter (mm)	54.1 (37; 70)	55 (40; 68)	0.68

LA, left atrium; PVI, Pulmonary vein isolation; TIA, transient ischemic attack.

Significantly shorter procedure times were observed in the Zero group compared to standard procedures (74.6 ± 13.2 min vs. 59.6 ± 10.7 min; p < 0.0001). Upon segmental analysis, this difference was primarily driven by a shorter access time associated with zero-fluoroscopy procedures (31 (23; 34.5) min vs. 17 (16; 20) min; p < 0.001), while other procedural segments and left atrial dwelling time (41.5 (36; 52.5) min vs. 40.5 (35; 46) min, p = 0.14) did not exhibit significant differences between the groups.

As anticipated, fluoroscopy time (132 (100; 160) sec vs. 0 (0; 0) sec, p < 0.0001) and fluoroscopy exposure (4.8 (4.1; 8.2) mGy vs. 0 (0; 0) mGy, p < 0.0001) were higher in the Standard group. No differences were found in terms of the number of RF applications (81 (73; 103) vs. 83 (71; 91), p = 0.67), total ablation time (1274 ± 290 s vs. 1221 ± 245 s, p = 0.17), or total ablation energy (54,986 ± 13,093 Ws vs. 55,500 ± 11,907 Ws, p = 0.48).

In all 100 cases, successful isolation of PVs was achieved, meeting the procedural endpoint and resulting in a 100% acute procedural success rate. Within the Zero group, all procedures were accomplished successfully without the use of fluoroscopy. The rate of first-pass isolation exhibited no significant difference between the Standard and Zero groups for both left-sided PVs (96% vs. 94%, p = 0.65) and right-sided PVs (90% vs. 86%, p = 0.54). No major complications occurred during the study period. Our comprehensive results are presented in Table 2.

**Table 2:** Summary of outcomes of procedural data.

	Standard group (n = 50)	Zero group (n = 50)	p
Total procedure time (min)	74.6 ± 13.2	59.6 ± 10.7	< 0.0001
Access time (min)	31 (23; 34.5)	17 (16; 20)	< 0.001
Time from the beginning of LA mapping to ablation (min)	8 (6; 11)	7 (6; 9)	0.10
Time from ablation to validation (min)	32.7 ± 9.4	30.6 ± 6.9	0.10
Time from the start of validation to the end of validation (min)	2 (1; 4.3)	2 (1; 3)	0.63
LA dwelling time (min)	41.5 (36; 52.5)	40.5 (35; 46)	0.14
Total RF ablation time (sec)	1274 ± 290	1221 ± 245	0.17
Total RF ablation energy (J)	54,986 ± 13,093	55,500 ± 11,097	0.48
RF application number (n)	81 (73; 103)	83 (71; 91)	0.67
Total fluoroscopy time (sec)	132 (100; 160)	0 (0; 0)	< 0.0001
Total fluoroscopy dose (mGy)	4.8 (4.1; 8.2)	0 (0; 0)	< 0.0001
Left-sided PVs first pass isolation (%)	48 (96)	47 (94)	0.65
Right-sided PVs first pass isolation (%)	45 (90)	43 (86)	0.54
Acute success (%)	50 (100)	50 (100)	1.0
Complication (%)	0	0	NA

LA, left atrium; NA, not applicable; PV, pulmonary veins; PVI, pulmonary vein isolation; RF, radiofrequency;

## Discussion

In our prospective, single-center observational trial, we validated the safety and feasibility of zero-fluoroscopy PVI procedures, even without experience in fluoroless AF ablation. Furthermore, the zero-fluoroscopy strategy was linked to a shorter procedural time, primarily attributed to the reduced time required from femoral vein puncture to achieve left atrial access. All procedures in the Zero group were successfully performed without the use of fluoroscopy, and no major complications were encountered.

Conventionally, in EP procedures, fluoroscopy assumes a pivotal role as the primary tool for guiding catheter placement, contributing substantially to approximately 95% of the overall fluoroscopy duration. Radiation exposure levels vary depending on the specific type of ablation procedures performed, with those involving AF ablations tending to yield the highest doses. Patients undergoing AF ablation may encounter an average radiation dose of 15 mSv per procedure, equivalent to the impact of approximately 750 chest X-rays.^12^ Individuals with obesity face an increased risk, as they are exposed to a double effective dose of radiation compared to individuals of average size. Furthermore, there is a notable occupational concern for electrophysiologists and medical staffs, given their estimated lifetime risk of cancer development resulting from radiation exposure, which stands at 0.5%^13^. Furthermore, the utilization of heavy lead aprons may lead to joint pain and associated health issues, adding to the significance of these concerns^7^.

Given these considerations, all invasive catheterization laboratories adhere to the ALARA principle, aiming to minimize the radiation dose “As Low As Reasonably Achievable.” This approach is implemented to safeguard both patients and medical staff.^14^.

Over the last two decades, significant technological advancements have transformed the field of cardiac EP. Currently, EAMSs and ICE stand out as reliable alternatives for visualizing catheter positions, offering viable options to the conventional use of fluoroscopy.

The integration of EAMSs has led to a considerable reduction in radiation exposure, resulting in reduced fluoroscopy exposure during catheter ablation and making fluoroless ablations a feasible and available option^6,15^. In contemporary clinical practice, visualizable (by EAMS) steerable sheaths have become accessible. Their utilization demonstrates advantages in reducing fluoroscopy and facilitating the accomplishment of fluoroscopy-free AF ablations^16,17^.

Emphasizing the utilization of zero-fluoroscopy guidance over the conventional fluoroscopic approach holds significant importance, especially in populations at higher risk, such as pregnant women and children^18–20^.

In AF ablations, the TSP crucial for gaining access to the left atrial septum, is essential. ICE is employed to facilitate a fluoroless transseptal puncture which is instrumental for real-time procedural visualization. Significantly, ICE excels in offering a clear perspective on the intricate and variable anatomy of the left atrium and in identifying potential thrombus formations within the left atrium. This capability not only enhances procedural precision but also plays a pivotal role in ensuring the safety and efficacy of the overall procedure^21^.

Prior data provide evidence regarding safety and feasibility of fluoroless TSP^22,23^. In our study, all TSPs in the Zero group were successfully executed without the need for fluoroscopy, even though the operator lacked relevant prior experience in this approach. However, it is crucial to recognize that this achievement may be attributed to the routine use of ICE in catheter ablations at our tertiary academic center. Our considerable experience in visualizing anatomical structures and tracking catheter manipulations with ICE likely contributed to the successful performance of TSPs without fluoroscopy.

Previous studies showed that zero-fluoroscopy strategy had no impact on procedure time compared to standard technique^7,24–26^. Lurie et al., reported shorter procedural times in patients undergoing AF ablation with the zero-fluoro strategy compared to standard technique. However, upon excluding redo cases from the analysis, no significant difference was observed between the groups^27^. Nevertheless, a recently published meta-analysis demonstrated a significant reduction in procedural time associated with the zero-fluoroscopy strategy in patients who underwent AF ablation^6^.

In our trial, our objective was to comprehensively analyze the impact of the zero-fluoroscopy technique on procedural time. To achieve this, we divided the procedure time into four fundamental segments, as outlined in the “Methods” section. The observed reduction in procedure time in the Zero group was primarily attributed to the shorter “access time,” defined as the duration from femoral vein puncture to the initiation of electroanatomical mapping.

Interestingly, the lack of prior experience with fluoroless TSP did not have a significant impact on this segment. The variation in access time between the groups could be attributed to the distinct catheter setup employed in the Zero and Standard groups. Specifically, in the Standard group, we utilized a decapolar catheter as an anatomical landmark for fluoroscopy plus ICE-guided TSP, necessitating additional femoral vein punctures. Furthermore, according to our institutional protocol in such cases, both right and left-sided femoral vein punctures were performed. Moreover, the positioning of the decapolar catheter was mandatory, and this task was carried out by EP fellows, potentially leading to an extended duration of this phase.

## Limitations

Several limitations need to be acknowledged in this study. Firstly, it was a nonrandomized, single-center observational trial with a limited number of included patients. Since all procedures were performed by a single electrophysiologist, the results cannot be generalized beyond the specific operator's expertise. Exclusion criteria, such as the omission of redo cases may limit the applicability of the results to certain patient populations, potentially impacting the external validity of the study. The conventional group required bilateral femoral vein punctures to insert three sheaths, compared to the single-sided puncture in the zero-fluoroscopy group, which only required two sheaths. Additionally, the use of a decapolar catheter as an anatomical marker in the conventional group added complexity and time to the procedure. Lastly, all preliminary steps before the transseptal puncture were performed by EP fellows, and their learning curve might have contributed to the longer access times in the conventional setup. Due to the differing catheter setups and the involvement of EP fellows, it cannot be conclusively stated that, under identical conditions and with expert operators, the zero-fluoroscopy approach is shorter compared to the conventional approach. To prove this, a randomized study would be desirable.

Addressing these limitations is crucial for fostering a more comprehensive understanding of the implications and generalizability of implementing zero-fluoroscopy techniques in AF ablation procedures.

## Conclusion

In our single-center trial involving 100 consecutive patients undergoing PVI for paroxysmal or persistent AF, we demonstrated that zero-fluoroscopy PVI is feasible and safe, even without previous experience in zero PVI cases.

## Data Availability

The datasets presented in this article are not readily available because of Hungarian legal regulations. Requests to access the datasets should be directed to PK, peter.kupo@gmail.com.

## References

[1] Hindricks G, Potpara T, Dagres N (2021). 2020 ESC Guidelines for the diagnosis and management of atrial fibrillation developed in collaboration with the European Association for Cardio-Thoracic Surgery (EACTS). Eur. Heart J..

[2] Chen C, Zhou X, Zhu M (2018). Catheter ablation versus medical therapy for patients with persistent atrial fibrillation: A systematic review and meta-analysis of evidence from randomized controlled trials. J. Intervent. Cardiac Electrophysiol..

[3] Morillo CA, Verma A, Connolly SJ (2014). Radiofrequency ablation vs antiarrhythmic drugs as first-line treatment of paroxysmal atrial fibrillation (RAAFT-2): A randomized trial. JAMA.

[4] Mark DB, Anstrom KJ, Sheng S (2019). Effect of catheter ablation vs medical therapy on quality of life among patients with atrial fibrillation: The CABANA randomized clinical trial. JAMA.

[5] Packer DL, Mark DB, Robb RA (2019). Effect of catheter ablation vs antiarrhythmic drug therapy on mortality, stroke, bleeding, and cardiac arrest among patients with atrial fibrillation: The CABANA randomized clinical trial. JAMA.

[6] Debreceni D, Janosi K, Bocz B (2023). Zero fluoroscopy catheter ablation for atrial fibrillation: A systematic review and meta-analysis. Front. Cardiovasc. Med..

[7] Zei P, Quadros K, Clopton P (2020). Safety and efficacy of minimal- versus zero-fluoroscopy radiofrequency catheter ablation for atrial fibrillation: A multicenter, prospective study. J. Innov. Cardiac Rhythm Manage..

[8] Fadhle A, Hu M, Wang Y (2020). The safety and efficacy of zero-fluoroscopy ablation versus conventional ablation in patients with supraventricular tachycardia. Kardiol. Pol..

[9] Chen G, Wang Y, Proietti R (2020). Zero-fluoroscopy approach for ablation of supraventricular tachycardia using the Ensite NavX system: A multicenter experience. BMC Cardiovasc. Disord..

[10] Debreceni D, Janosi K, Vamos M, Komocsi A, Simor T, Kupo P (2022). Zero and minimal fluoroscopic approaches during ablation of supraventricular tachycardias: A systematic review and meta-analysis. Front. Cardiovasc. Med..

[11] Mehran R, Rao SV, Bhatt DL (2011). Standardized bleeding definitions for cardiovascular clinical trials: A consensus report from the bleeding academic research consortium. Circulation.

[12] Heidbuchel H, Wittkampf FHM, Vano E (2014). Practical ways to reduce radiation dose for patients and staff during device implantations and electrophysiological procedures. Europace.

[13] Venneri L, Rossi F, Botto N (2009). Cancer risk from professional exposure in staff working in cardiac catheterization laboratory: Insights from the National Research Council’s Biological Effects of Ionizing Radiation VII Report. Am. Heart J..

[14] Scaglione M, Ebrille E, Di CF, Gaita F, Bradfield JS (2015). Catheter ablation of atrial fibrillation without radiation exposure using a 3D mapping system. J. Atr. Fibrill..

[15] Gaita F, Guerra PG, Battaglia A, Anselmino M (2016). The dream of near-zero X-rays ablation comes true. Eur. Heart J..

[16] Fitzpatrick N, Mittal A, Galvin J (2023). The impact of steerable sheath visualization during catheter ablation for atrial fibrillation. Europace.

[17] Janosi K, Debreceni D, Janosa B, Bocz B, Simor T, Kupo P (2022). Visualizable vs standard, non-visualizable steerable sheath for pulmonary vein isolation procedures: Randomized, single-centre trial. Front. Cardiovasc. Med..

[18] Driver K, Chisholm CA, Darby AE, Malhotra R, Dimarco JP, Ferguson JD (2015). Catheter ablation of arrhythmia during pregnancy. J. Cardiovasc. Electrophysiol..

[19] Drago F, Silvetti MS, Di Pino A, Grutter G, Bevilacqua M, Leibovich S (2002). Exclusion of fluoroscopy during ablation treatment of right accessory pathway in children. J. Cardiovasc. Electrophysiol..

[20] Brugada J, Katritsis DG, Arbelo E (2020). 2019 ESC Guidelines for the management of patients with supraventricular tachycardiaThe Task Force for the management of patients with supraventricular tachycardia of the European Society of Cardiology (ESC). Eur. Heart J..

[21] Antolič B, Kajdič N, Vrbajnščak M, Jan M, Žižek D (2021). Integrated 3D intracardiac ultrasound imaging with detailed pulmonary vein delineation guided fluoroless ablation of atrial fibrillation. Pacing Clin. Electrophysiol..

[22] Baykaner T, Quadros KK, Thosani A (2020). Safety and efficacy of zero fluoroscopy transseptal puncture with different approaches. Pacing Clin. Electrophysiol..

[23] Žižek D, Antolič B, Prolič Kalinšek T (2021). Intracardiac echocardiography-guided transseptal puncture for fluoroless catheter ablation of left-sided tachycardias. J. Interv. Card. Electrophysiol..

[24] Bulava A, Hanis J, Eisenberger M (2015). Catheter ablation of atrial fibrillation using zero-fluoroscopy technique: A randomized trial. Pacing Clin. Electrophysiol..

[25] Elvin Gul E, Azizi Z, Alipour P (2021). Fluoroless catheter ablation of atrial fibrillation: Integration of intracardiac echocardiography and cartosound module. J. Atr. Fibrill..

[26] Lyan E, Tsyganov A, Abdrahmanov A (2018). Nonfluoroscopic catheter ablation of paroxysmal atrial fibrillation. Pacing. Clin. Electrophysiol..

[27] Lurie A, Amit G, Divakaramenon S, Acosta JG, Healey JS, Wong JA (2021). Outcomes and safety of fluoroless catheter ablation for atrial fibrillation. CJC Open.

